# Targeting SOX2 as a Therapeutic Strategy in Glioblastoma

**DOI:** 10.3389/fonc.2016.00222

**Published:** 2016-10-24

**Authors:** Laura Garros-Regulez, Idoia Garcia, Estefania Carrasco-Garcia, Aquilino Lantero, Paula Aldaz, Leire Moreno-Cugnon, Olatz Arrizabalaga, Jose Undabeitia, Sergio Torres-Bayona, Jorge Villanua, Irune Ruiz, Larraitz Egaña, Nicolas Sampron, Ander Matheu

**Affiliations:** ^1^Cellular Oncology Group, Department of Oncology, Biodonostia Institute, San Sebastian, Spain; ^2^IKERBASQUE Foundation, Bilbao, Spain; ^3^Opioid Research Group, Department of Pharmaceutical Chemistry, University of Innsbruck, Innsbruck, Austria; ^4^Neuro-Oncology Committee, Donostia Hospital, San Sebastian, Spain

**Keywords:** SOX2, glioblastoma, tumor-initiating cells, temozolomide resistance, glioma stem cells, self-renewal inhibition

## Abstract

Glioblastoma is the most common and malignant brain cancer in adults. Current therapy consisting of surgery followed by radiation and temozolomide has a moderate success rate and the tumor reappears. Among the features that a cancer cell must have to survive the therapeutic treatment and reconstitute the tumor is the ability of self-renewal. Therefore, it is vital to identify the molecular mechanisms that regulate this activity. Sex-determining region Y (SRY)-box 2 (SOX2) is a transcription factor whose activity has been associated with the maintenance of the undifferentiated state of cancer stem cells in several tissues, including the brain. Several groups have detected increased SOX2 levels in biopsies of glioblastoma patients, with the highest levels associated with poor outcome. Therefore, SOX2 silencing might be a novel therapeutic approach to combat cancer and particularly brain tumors. In this review, we will summarize the current knowledge about SOX2 in glioblastoma and recapitulate several strategies that have recently been described targeting SOX2 in this malignancy.

## SOX2

Sex-determining region Y (SRY)-box (SOX) factors are a family of transcriptional regulators that carry out important functions during embryonic development and which are key components for the maintenance of the stem cells in adult tissues. This family of transcription factors is characterized by a conserved high mobility group (HMG) DNA-binding domain and is composed of 20 members divided into 8 groups (from A to H), based on their HMG sequence identity (1). Members within a subfamily conserve at least 80% identity in their HMG-domain, in addition to sharing other well-conserved regions. Moreover, members from the same group might have overlapping expression patterns, share biochemical properties, and perform synergistic or redundant functions. By contrast, members from different subgroups usually develop different functions (2).

SOX2 is a member of the SOXB1 group (together with SOX1 and SOX3), which is required for the maintenance of the embryo before implantation. SOX2 has a role in cell fate and maintenance of the progenitors’s identity during embryogenesis. It is also important for tissue homeostasis and regeneration by maintaining stem cell activity in several compartments, particularly in the central nervous system (CNS), in adults (3). During recent years, several studies have demonstrated the impact of SOX2 deregulation in a wide variety of human diseases. A heterozygous inactivation of *SOX2* causes syndromic microphthalmia-3 (MCOPS3), a genetic disease characterized by anophthalmia, microphthalmia mild hypopituitarism, and sometimes learning difficulties, convulsions, motor dysfunctions, and growth problems (4). On the contrary, SOX2 upregulation has been linked to the development and maintenance of several types of cancers (3, 5–7).

## Glioblastoma

Tumors of the CNS form a heterogeneous group of diseases that comprise less than 2% of the total number of cancer cases. Every year in the world, ~350,000 people are diagnosed with gliomas, making it the most common primary brain tumor (8). According to clinical and histopathological characteristics, WHO classified them by four grades of malignancy: pylocitic astrocytoma (grade I); diffuse astrocytoma (grade II); anaplastic astrocytoma (grade III), and glioblastoma multiforme (GBM, grade IV). GBM is the most common, malignant, and lethal glioma subtype in adults accounting for 12–15% of all brain tumors and about 50% of gliomas. The incidence ranges from 1 to 5 cases per 100,000 people per year, with an average patient survival of around 15 months (8). This prognosis identifies this type of tumor as one of the most aggressive and fatal cancers overall. According to the clinical presentation, there are two main GBM subtypes: primary or *de novo* GBM and secondary GBM. Primary tumors, the most common form, typically appear in older patients without any prior clinical or histological evidence of a lower grade precursor lesion and they have an aggressive clinical course. Secondary tumors are more frequent in younger people and they progress from a previous lower grade glioma with a less aggressive clinical course (9). Genetic and transcriptomic expression studies have allowed a more detailed molecular classification identifying four GBM subtypes: (i) classical, with *EGFR* amplification and overexpression, *CDKN2A* and *PTEN* deletion, *NES* overexpression and activation of NOTCH and SHH signaling pathways; (ii) mesenchymal, with loss of *NF1, TP53*, and *PTEN*, overexpression of *MET, CHI3L1, CD44*, and *MERTK*, and activation of the TNF and NF-kB pathways; (iii) proneural, with *PDGFR* amplification, loss or mutation of *IDH1, PIK3K, TP53, CDKN2A*, and *PTEN*, and activation of HIF, PI3K, and PDGFR pathways; and (iv) neural, with *EGFR* amplification and overexpression, and expression of neuronal markers, such as *NEFL, SYT1*, and/or *GABRA1* (10). Different subtypes are associated with variable prognosis and response to therapy, and this heterogeneity and this therapy resistance are likely the main characteristics responsible for the glioblastoma patients’ dismal outcome.

**Figure 1:**
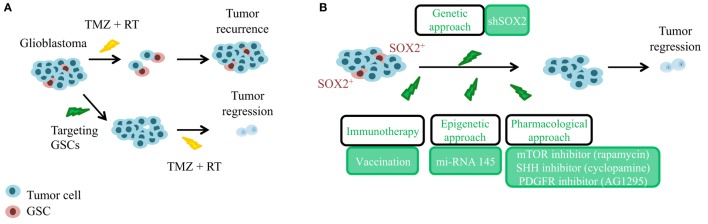
**Significance of glioma stem cells (GSCs) and SOX2 targeting in the recurrence of glioblastoma**. **(A)** Glioblastoma is a heterogeneous tumor composed of GSCs (in red, chemo-, and radiotherapy resistant) and of differentiated tumor cells (in blue, chemo-, and radiotherapy responsive). GSCs need to be targeted before current standard approaches to achieve tumor regression. TMZ, temozolomide; RT, radiotherapy. **(B)** In order to avoid tumor recurrence, genetic, epigenetic, and pharmacological approaches targeting GSCs expressing high levels of SOX2 have been postulated.

The cancer stem cell (CSC) theory postulates a hierarchically organized system in opposition to the stochastic model of tumor growth. The CSC model suggests that only a small group of cells have quiescence and self-renewal capacity within the tumor bulk, and that those are responsible for tumor maintenance and recurrence (11). Nowadays, there is a lot of evidence that supports the existence of CSCs in GBM, called glioma stem cells (GSC), and their relevance in the etiology of GBM. Several groups have been able to isolate GSCs from patient-derived tumors and multiple experimental data have shown that these cells are responsible for glioblastoma initiation and maintenance (12, 13), as well as for recurrence and chemoresistance (14–16). Moreover, there are different explanations for their origin. One of them proposes that neural stem cells (NSCs) undergo malignant transformation while retaining stem cell features. Indeed, inactivation of *TP53, INK4a/ARF* locus, *PTEN*, and *NF1* tumor suppressors or activation of EGFR/PDGFR/PI3K oncogenic pathways in NSCs induces high-grade gliomas (17). Similarly, transient amplifying progenitors have been also shown as GSCs and the cell of origin of GBM (18). Another theory claims that more mature or differentiated cells are reprogrammed and form GSCs and high-grade gliomas. Indeed, several mutations in astrocytes, oligodendrocyte progenitors, or in neurons are sufficient to confer stem cell properties during neoplastic transformation (19, 20). Therefore, in order to establish efficient treatments that can induce a long-lasting clinical response in GBM, it is important to develop strategies that can specifically target GSCs (Figure 1A). CSCs, including GSCs, achieve self-renewal through asymmetric division, in which one daughter cell retains the self-renewal ability, and the other is directed to differentiation. Moreover, heterogeneous tumor cell populations and their respective cell division mode have been shown to confer differential sensitivity to therapy in brain tumors (21). Therefore, modulation of asymmetric and symmetric division of GSCs may provide novel strategies for targeting differentially the GSC and the bulk tumor mass. Several drugs and approaches have been postulated (22) to directly target the GSCs population and/or the molecular mechanisms underlying their regulation. In this review, we focused our attention on targeting *SOX2* gene.

## SOX2 Activity and GBM

Several studies have identified an overexpression of SOX2 in GBM patient samples. It was first found elevated in 90% of human biopsies studied at the mRNA and protein level in 2007. This research also showed that SOX2 expression was restricted to the nucleus (23). Since then, overexpression of SOX2 (with varying percentages of positive cases) was observed in several different and independent cohorts (16, 24–27). Importantly, high levels of SOX2 have been associated with tumor aggressiveness and worse prognosis (28, 29). Moreover, several groups, including ours, identified SOX2 enrichment in the undifferentiated GSC populations and demonstrated that SOX2 possesses an important role in the maintenance of GSCs. Indeed, downregulation of SOX2 through RNA interference strategies in GSCs impairs proliferation and their ability to form tumors *in vivo* (25, 30–32). Additionally, silencing of SOX2 leads to reduced migration and invasion capabilities (25, 33), while it increases senescence and produces an arrest of the cell cycle in G0/G1 (16, 34). The impact of SOX2 in glioblastoma cells has been further substantiated with overexpression studies. Indeed, ectopic elevation of SOX2 increases the capacity of invasion and migration (25), in addition to cell proliferation and self-renewal activity in conventional glioma cell lines (16). In agreement with this last function, SOX2 is one of the transcription factors, together with POU3F2, OLIG2, and SALL2, which is sufficient to reprogram differentiated glioma cells into induced GSCs, similar to GSCs obtained from human biopsies (35). Altogether these data show that glioma cells have a dependence on SOX2 to maintain their tumorigenic activity with GSCs displaying high levels of SOX2. They also demonstrate that SOX2 possesses an important role in the maintenance of GSCs.

In regard to a putative role of SOX2 controlling cell division modes, a recent work showed that the inhibition of the FACT chaperone complex in GSCs promotes their asymmetrical division in a process that involves SOX2 downregulation (36). In line with these results, other authors found that the knockdown of HMGA1, a chromatin structure regulator, not only in GSCs but also in colon CSCs, induces an asymmetric division together with a decrease in SOX2 expression (37, 38). These works suggest that SOX2 action in the maintenance of undifferentiated GSCs could rely on effects promoting symmetrical in addition to the expected asymmetrical division. Importantly, these results support a hierarchical model of glioma cells controlled by SOX2 expression, which brings up the idea to target *SOX2* or to find downstream targetable genes as a strategy to eliminate GSCs and subsequently the tumor.

## Upstream Regulation of SOX2

The regulation of SOX2 is a complex network of transcriptional, post-transcriptional, and post-translational regulators (Table 1). Some of these regulators are altered in GBM and lead to the overexpression of SOX2. Four main signaling pathways are involved in SOX2 expression, including TGF-β, SHH, EGFR, and FGFR. All these signaling pathways are aberrantly activated in GBM, which leads to the maintenance of the tumor at least in part through SOX2 factor overexpression. The inhibition of TGF-β signaling decreases the tumorigenicity of GSCs by the suppression of SOX2 activity (31). SOX2 function is also mediated by other members of the SOX family, such as SOX4, acting downstream of the TGF-β signaling pathway, and forming a complex with OCT4 at the *SOX2* promoter (31, 39). SHH pathway is initiated with the binding of SHH ligand to PTCH receptor, causing the activation of SMO. Active SMO will activate GLI1/2, which then translocates into the nucleus and activates SOX2. The regulation of SOX2 by SHH occurs in neural and brain stem cells (40, 41), and the pharmacological inhibition of these pathways silences SOX2 expression and impairs glioma cells’ tumorigenic activity (16). The FGFR pathway regulates SOX2 expression through two main signaling cascades: (i) MEK/ERK and (ii) PI3K/AKT/mTOR, two signaling pathways activated in GBM, and whose suppression leads to the inhibition of tumorigenesis and self-renewal of GSCs (42). The MEK/ERK pathway regulates the expression of SOX2 through the final phosphorylation of ERK, which translocates into the nucleus and activates the transcription of SOX2. The PI3K/AKT/mTOR pathway regulates positively the expression of SOX2 through the activation of the mammalian target of rapamycin complex 1 (mTORC1). The inhibition of mTORC1 by rapamycin in GSCs leads to an inhibition of the SOX2 expression and a decrease in self-renewal activity (16). SOX2 is also regulated by EGFRvIII, a frequent mutant in GBM that leads to the activation of pro-oncogenic signaling in GBM. Indeed, the expression of EGFRvIII positively correlates with the expression of SOX2 and is associated with an enhanced self-renewal ability and tumor-initiating activity (43). This correlation has demonstrated that it is carried out by the axis EGFRvIII–STAT3–PEDF–Notch (44).

**Table 1:** **Summary of relevant findings of SOX2 in glioblastoma**.

Year	Finding	Reference
2007	SOX2 is overexpressed in human glioma samples	Schmitz et al. (23)
2009	Genetic SOX2 silencing impairs GSCs activity	Gangemi et al. (30)
2011	Identification of SOX2 downstream targets in GBM with miRNA145–SOX2 feedback loop	Fang et al. (48)
2011	Genetic and epigenetic regulation of SOX2 in GBM samples	Alonso et al. (25)
2011	Identification of SOX2 as a target for combination treatments with tyrosine-kinase inhibitors	Hägerstrand et al. (32)
2011	Identification of SOX2 downstream of TGF-β signaling in GSCs	Ikushima et al. (31)
2014	Elevated SOX2 promotes dedifferentiation and acquisition of GSC characteristics in GBM cells	Suvà et al. (35)
2007 and 2014	First results supporting SOX2 mediated immunotherapy in mouse models and human samples	Schmitz et al. (23)
		Favaro et al. (41)
2016	SOX2 induces chemoresistance, which is inhibited by rapamycin	Garros-Regulez et al. (16)

SOX2 can also be regulated via post-translational modifications, such as ubiquitination, phosphorylation, and acetylation. Acetylation and phosphorylation enhance the export of SOX2 to the cytoplasm and inhibit the ability to bind DNA in embryonic stem cells (45, 46). By contrast, the phosphorylation of SOX2 by AKT stabilizes the protein and enhances the transcriptional activity of SOX2 (47). However, the function of these modifications in GSCs’ activity remains elusive.

Additionally, *SOX2* genetic amplification and promoter DNA hypomethylation has been identified in a set of GBM patients, further expanding the mechanism responsible for SOX2 upregulation in glioblastoma samples and GSCs (25). Moreover, the regulation of SOX2 through different miRNAs, including miRNA21 or miRNA145, has been described in glioma cells, with this axis having relevant functions in GSCs’ activity and in the clinic (29, 48).

## Downstream Regulation of SOX2

Several studies have started to characterize downstream targets of SOX2 in glioblastoma. A study of Fang et al. identified SOX2 downstream targets by ChIP-seq and microarray analyses in the LN229 glioma cell line. They found 4,883 SOX2 binding regions in the GBM cancer genome. Moreover, they detected 489 genes whose expression was altered with SOX2 inhibition, including additional SOX family members, cytokines, or BEX members with tumor suppressor activity in glioblastoma. They also identified 105 pre-miRNAs (corresponding to 95 mature miRNAs) that were differentially expressed in SOX2 knockdown glioblastoma cells. Among them, they observed that miRNA145 and SOX2 form a double-negative feedback loop in GBM cells (48), demonstrating that the relationship between SOX2 and miRNA is bidirectional. We have recently observed that several oncogenic SOX2 functions are mediated by SOX9, another member of the SOX family (16), which also carries out important functions in GSC regulation and glioblastoma (26). This regulation occurs at post-transcriptional level (16). Additionally, specific phenotypes associated with SOX2 have been linked to different genes and signaling pathways. Indeed, SOX2-regulated migratory and invasive capacities are mediated by RhoA-dependent pathway and focal adhesion kinase (FAK) signaling, whereas proliferation is mediated by CYCLIN D1 expression (34). WNT signaling pathway, self-renewal, and retinoic acid associated genes are within the genes involved in SOX2-mediated glioma cell plasticity and astrocytic differentiation (49).

## Therapeutic Approaches toward SOX2 Reduction in GBM

The current chemotherapeutic agent for newly diagnosed GBM is temozolomide (TMZ), which extends patient survival from 12 to 15 months (50). A role for SOX2 in TMZ chemoresistance has been deciphered during recent years. Thus, cells with elevated SOX2 expression are more resilient to TMZ, whereas its inhibition sensitizes glioma cells to this agent (16). This cellular finding is correlated with clinical information. High levels of SOX2 have been included as a marker of the proneural subtype, which has been shown to be the most resistant subgroup to current therapeutic radio- and chemotherapy treatment (10). The involvement of SOX2 in chemoresistance has been further substantiated through different mechanisms. The inhibition of *SOX2* by miRNA145 decreases the chemoresistance of GSCs and increases the sensitivity to radiation and TMZ (51). The overexpression of ID-4 suppresses the expression of miRNA9, that can repress *SOX2*, leading to an increase in the SOX2 expression. SOX2 induction enhances the ATP-binding cassette (ABC) transporters, ABCC3 and ABCC6, through direct transcriptional regulation. The activation of ABC transporters confers chemoresistance to GSCs (52). These facts together with its prominent role in the regulation of GSCs suggest that SOX2 might be a key responsible factor for resistance to current chemotherapy and postulate that targeting its activity may offer a novel, attractive therapeutic approach to treat glioblastoma patients.

Several strategies are starting to use SOX2 directly or indirectly to target GSCs (Figure 1B). PDGFR signaling has been involved in glioblastoma biology through studies based on analyses of human tumor tissue, cultured glioblastoma cells, and mouse glioblastoma models (22). Similarly, IGF1-R signaling has been described in glioblastoma, and findings from preclinical studies suggest favorable combination effects when IGF1-R inhibitors have been combined with other receptor tyrosine-kinase (RTK)-targeting agents. Interestingly, a combination therapy with PDGF and IGF1 receptor inhibitors (imatinib and NVP-AEW541) produces a significant tumor growth reduction through SOX2 downregulation and GSC sensitization (32).

Rapamycin is an allosteric inhibitor of mTOR, which has been shown to dramatically reduce the self-renewal and tumorigenic activity of glioma cells and GSCs (53). In agreement with these results, several phase I and II clinical trials, with some of its rapalogs, such as everolimus or temsirolimus, alone or in combination, showed radiographic and symptomatic evidence of improvement in delaying tumor progression without provoking high toxicity in patients with newly diagnosed or recurrent glioblastoma (54). It has recently been demonstrated that mTOR regulates the expression of SOX2. Genetic mTOR silencing or pharmacological treatment with rapamycin markedly reduced SOX2 levels in glioma cells (16). Interestingly, the same work showed that the combination of rapamycin and TMZ was more efficient and displayed increased cytotoxicity in cells with high endogenous SOX2 levels (16). Cyclopamine, an inhibitor of the SHH pathway, was also proved effective in reducing SOX2 expression and inducing cytotoxicity in *in vitro* studies, but unlike rapamycin, its combination with TMZ did not increase the sensitivity of glioma cells to chemotherapy (16). Furthermore, several studies have demonstrated the capability of the GSCs to transdifferentiate into tumor-derived vascular endothelial and mural-like cells in a VEGF-independent manner, making the tumor resistant to anti-vascular therapy (55, 56). This transdifferentiation relies on high levels of NESTIN and CD133 stem markers; however, SOX2 has not been evaluated. It would be of interest to determine the role of SOX2 in transdifferentiation and if knocking down its expression levels is sufficient to prevent the process.

Alternative treatments are arising using SOX2 protein as the principal target of new therapies. Immunotherapy represents a promising treatment option to improve the clinical outcome of patients suffering from GBM. In 2014, Dr Nicolis’ group did transplants of GSCs in mice brains using peptide vaccination against SOX2. Peptide vaccination alone increased the mice’ survival and the vaccination in combination with the current treatment with TMZ doubled the mice’ survival time (41). Before, Schmitz and coworkers identified SOX2 as a glioma-associated antigen abundantly and specifically overexpressed in glioma cells. In addition, they identified an immunogenic HLA-A*0201-restricted T-cell epitope derived from SOX2 that effectively activated tumor-directed cytotoxic T lymphocytes (23). These results highlight the suitability of SOX2 for a novel strategy based in immunotherapy in monotherapy or in combination with current therapies for the treatment of glioblastoma patients.

The use of miRNA delivery could be another therapy linked to SOX2 for cancer cells in the brain. As described above, miRNA145 is associated with SOX2. Interestingly, miRNA145 delivery in GSCs and in xenograft studies *in vivo* demonstrated the ability to suppress the tumorigenicity by direct downregulation of SOX2 protein with cells becoming more sensitive to chemotherapeutic agents, such as TMZ or cisplatin (51). The promising therapeutic prospect of miR-145 might improve current cancer treatments, especially for those tumors that have developed a resistance to conventional therapeutic methods. However, a note of caution needs to be included since the use of viral vectors for gene delivery may be accompanied by several problems, including an immune response.

## Concluding Remarks

Glioblastoma is the most common and malignant adult primary brain tumor with a dismal patient prognosis. It is characterized by presenting significant heterogeneity at the genetic, molecular, cellular, and morphological level, which severely affects clinical practice. Tumor bulk is formed by differentiated cells targetable with chemo- and radiotherapy and GSCs, which need to be eradicated in order to achieve effective therapeutic response.

Sex-determining region Y (SRY)-box 2 transcription factor is important during embryonic development and for the maintenance of stem cell properties of the CNS in adult and aged stages (3, 57, 58). It is also a key regulator of stemness in CSCs and its biological function has been widely described in GBM, associated with stemness activity and a poor clinical outcome (Table 1). Therefore, SOX2 is a strong molecular candidate to be targeted in glioblastoma samples and the potential benefit of SOX2 targeting in the scenario of such a lethal tumor is not negligible. However, a point to take into account with regard to SOX2 targeting for GBM treatment is that other SOX2 expressing cell populations as astrocytes, which develop relevant roles in neurogenesis, could be susceptible to the treatments. The consequences of this must be evaluated and other molecules acting downstream of SOX2, and more specific of GSCs, should be identified and considered as targets.

Transcriptional regulation and/or post-transcriptional suppression of SOX2 through miRNA regulation are promising approaches. There are also encouraging results with SOX2 immunotherapy or combining tyrosine-kinase and IGF1 inhibitors. These results show the preclinical proof of concept that silencing SOX2 activity is an effective strategy in glioblastoma. More solid and extensive preclinical results and clinical trials with the postulated combination of therapies in glioblastoma patients whose biopsies express elevated SOX2 are needed to establish their clinical impact.

## Author Contributions

LG-R and IG, first authors of the review, wrote most of the review. NS and AM, senior authors, designed the review, directed the work, and wrote the manuscript. The additional authors suggested ideas and revised the manuscript.

## Conflict of Interest Statement

The authors declare that the research was conducted in the absence of any commercial or financial relationships that could be construed as a potential conflict of interest.

## References

[1] Kamachi Y Kondoh H. Sox proteins: regulators of cell fate specification and differentiation. Development (2013) 140:4129–44. 10.1242/dev.091793 24086078

[2] Wegner M All purpose Sox: the many roles of Sox proteins in gene expression. Int J Biochem Cell Biol (2009) 42:381–90. 10.1016/j.biocel.2009.07.006 19631281

[3] Sarkar A Hochedlinger K. The sox family of transcription factors: versatile regulators of stem and progenitor cell fate. Cell Stem Cell (2013) 12:15–30. 10.1016/j.stem.2012.12.007 23290134 PMC3608206

[4] Kelberman D Rizzoti K Avilion A Bitner-Glindzicz M Cianfarani S Collins J Mutations within Sox2/SOX2 are associated with abnormalities in the hypothalamo-pituitary-gonadal axis in mice and humans. J Clin Invest (2006) 116:2442–55. 10.1172/JCI28658 16932809 PMC1551933

[5] Bass AJ Watanabe H Mermel CH Yu S Perner S Verhaak RG SOX2 is an amplified lineage-survival oncogene in lung and esophageal squamous cell carcinomas. Nat Genet (2009) 41:1238–42. 10.1038/ng.465 19801978 PMC2783775

[6] Li H Collado M Villasante A Matheu A Lynch CJ Cañamero M p27(Kip1) directly represses Sox2 during embryonic stem cell differentiation. Cell Stem Cell (2012) 11:845–52. 10.1016/j.stem.2012.09.014 23217425 PMC3549496

[7] Carrasco-Garcia E Santos JC Garcia I Brianti M García-Puga M Pedrazzoli J Jr Paradoxical role of SOX2 in gastric cancer. Am J Cancer Res (2016) 6:701–13. 27186426 PMC4859879

[8] Ostrom QT Bauchet L Davis FG Deltour I Fisher JL Langer CE The epidemiology of glioma in adults: a “state of the science” review. Neuro Oncol (2014) 16:896–913. 10.1093/neuonc/nou087 24842956 PMC4057143

[9] Ohgaki H Kleihues P. The definition of primary and secondary glioblastoma. Clin Cancer Res (2013) 19:764–72. 10.1158/1078-0432.CCR-12-3002 23209033

[10] Verhaak RG Hoadley KA Purdom E Wang V Qi Y Wilkerson MD Integrated genomic analysis identifies clinically relevant subtypes of glioblastoma characterized by abnormalities in PDGFRA, IDH1, EGFR, and NF1. Cancer Cell (2010) 17:98–110. 10.1016/j.ccr.2009.12.020 20129251 PMC2818769

[11] Pattabiraman DR Weinberg RA. Tackling the cancer stem cells – what challenges do they pose? Nat Rev Drug Discov (2014) 13:497–512. 10.1038/nrd4253 24981363 PMC4234172

[12] Galli R Binda E Orfanelli U Cipelletti B Gritti A De Vitis S Isolation and characterization of tumorigenic, stem-like neural precursors from human glioblastoma. Cancer Res (2004) 64:7011–21. 10.1158/0008-5472.CAN-04-1364 15466194

[13] Singh SK Hawkins C Clarke ID Squire JA Bayani J Hide T Identification of human brain tumour initiating cells. Nature (2004) 432:396–401. 10.1038/nature03128 15549107

[14] Bao S Wu Q McLendon RE Hao Y Shi Q Hjelmeland AB Glioma stem cells promote radioresistance by preferential activation of the DNA damage response. Nature (2006) 444:756–60. 10.1038/nature05236 17051156

[15] Chen J Li Y Yu TS McKay RM Burns DK Kernie SG A restricted cell population propagates glioblastoma growth after chemotherapy. Nature (2012) 488:522–6. 10.1038/nature11287 22854781 PMC3427400

[16] Garros-Regulez L Aldaz P Arrizabalaga O Moncho-Amor V Carrasco-Garcia E Manterola L mTOR inhibition decreases SOX2-SOX9 mediated glioma stem cell activity and temozolomide resistance. Expert Opin Ther Targets (2016) 20:393–405. 10.1517/14728222.2016.1151002 26878385 PMC4898154

[17] Alcantara Llaguno S Chen J Kwon CH Jackson EL Li Y Burns DK Malignant astrocytomas originate from neural stem/progenitor cells in a somatic tumor suppressor mouse model. Cancer Cell (2009) 15:45–56. 10.1016/j.ccr.2008.12.006 19111880 PMC2650425

[18] Alcantara Llaguno SR Wang Z Sun D Chen J Xu J Kim E Adult lineage-restricted CNS progenitors specify distinct glioblastoma subtypes. Cancer Cell (2015) 28:429–40. 10.1016/j.ccell.2015.09.007 26461091 PMC4607935

[19] Persson AI Petritsch C Swartling FJ Itsara M Sim FJ Auvergne R Non-stem cell origin for oligodendroglioma. Cancer Cell (2010) 18:669–82. 10.1016/j.ccr.2010.10.033 21156288 PMC3031116

[20] Friedmann-Morvinski D Bushong EA Ke E Soda Y Marumoto T Singer O Dedifferentiation of neurons and astrocytes by oncogenes can induce gliomas in mice. Science (2012) 338:1080–4. 10.1126/science.1226929 23087000 PMC3595315

[21] Lewis KM Petritsch C. Asymmetric cell division: implications for glioma development and treatment. Transl Neurosci (2013) 4:484–503. 10.2478/s13380-013-0148-8 25530875 PMC4269374

[22] Carrasco-Garcia E Sampron N Aldaz P Arrizabalaga O Villanua J Barrena C Therapeutic strategies targeting glioblastoma stem cells. Recent Pat Anticancer Drug Discov (2013) 8:216–27. 10.2174/15748928113089990002 23607282

[23] Schmitz M Temme A Senner V Ebner R Schwind S Stevanovic S Identification of SOX2 as a novel glioma-associated antigen and potential target for T cell-based immunotherapy. Br J Cancer (2007) 96:1293–301. 10.1038/sj.bjc.6603802 17375044 PMC2360145

[24] Annovazzi L Mellai M Caldera V Valente G Schiffer D. SOX2 expression and amplification in gliomas and glioma cell lines. Cancer Genomics Proteomics (2011) 8:139–47. 21518820

[25] Alonso MM Diez-Valle R Manterola L Rubio A Liu D Cortes-Santiago N Genetic and epigenetic modifications of Sox2 contribute to the invasive phenotype of malignant gliomas. PLoS One (2011) 6:e26740. 10.1371/journal.pone.0026740 22069467 PMC3206066

[26] de la Rocha AM Sampron N Alonso MM Matheu A. Role of SOX family of transcription factors in central nervous system tumors. Am J Cancer Res (2014) 4:312–24. 25057435 PMC4106650

[27] Brennan CW Verhaak RG McKenna A Campos B Noushmehr H Salama SR The somatic genomic landscape of glioblastoma. Cell (2013) 155:462–77. 10.1016/j.cell.2013.09.034 24120142 PMC3910500

[28] Ben-Porath I Thomson MW Carey VJ Ge R Bell GW Regev A An embryonic stem cell-like gene expression signature in poorly differentiated aggressive human tumors. Nat Genet (2008) 40:499–507. 10.1038/ng.127 18443585 PMC2912221

[29] Sathyan P Zinn PO Marisetty AL Liu B Kamal MM Singh SK Mir-21-Sox2 axis delineates glioblastoma subtypes with prognostic impact. J Neurosci (2015) 35:15097–112. 10.1523/JNEUROSCI.1265-15.2015 26558781 PMC4642241

[30] Gangemi RM Griffero F Marubbi D Perera M Capra MC Malatesta P SOX2 silencing in glioblastoma tumor-initiating cells causes stop of proliferation and loss of tumorigenicity. Stem Cells (2009) 27:40–8. 10.1634/stemcells.2008-0493 18948646

[31] Ikushima H Todo T Ino Y Takahashi M Miyazawa K Miyazono K. Autocrine TGF-beta signaling maintains tumorigenicity of glioma-initiating cells through Sry-related HMG-box factors. Cell Stem Cell (2009) 5:504–14. 10.1016/j.stem.2009.08.018 19896441

[32] Hägerstrand D He X Bradic Lindh M Hoefs S Hesselager G Ostman A Identification of a SOX2-dependent subset of tumor- and sphere-forming glioblastoma cells with a distinct tyrosine kinase inhibitor sensitivity profile. Neuro Oncol (2011) 13:1178–91. 10.1093/neuonc/nor113 21940738 PMC3199157

[33] Velpula KK Dasari VR Tsung AJ Dinh DH Rao JS. Cord blood stem cells revert glioma stem cell EMT by down regulating transcriptional activation of Sox2 and Twist1. Oncotarget (2011) 2:1028–42. 10.18632/oncotarget.367 22184289 PMC3282065

[34] Oppel F Müller N Schackert G Hendruschk S Martin D Geiger KD SOX2-RNAi attenuates S-phase entry and induces RhoA-dependent switch to protease-independent amoeboid migration in human glioma cells. Mol Cancer (2011) 10:137. 10.1186/1476-4598-10-137 22070920 PMC3228695

[35] Suvà ML Rheinbay E Gillespie SM Patel AP Wakimoto H Rabkin SD Reconstructing and reprogramming the tumor-propagating potential of glioblastoma stem-like cells. Cell (2014) 157:580–94. 10.1016/j.cell.2014.02.030 24726434 PMC4004670

[36] Dermawan JK Hitomi M Silver DJ Wu Q Sandlesh P Sloan AE Pharmacological targeting of the histone chaperone complex FACT preferentially eliminates glioblastoma stem cells and prolongs survival in preclinical models. Cancer Res (2016) 76:2432–42. 10.1158/0008-5472.CAN-15-2162 26921329 PMC4873320

[37] Colamaio M Tosti N Puca F Mari A Gattordo R Kuzay Y HMGA1 silencing reduces stemness and temozolomide resistance in glioblastoma stem cells. Expert Opin Ther Targets (2016) 20:1169–79. 10.1080/14728222.2016.1220543 27486901

[38] Puca F Colamaio M Federico A Gemei M Tosti N Bastos AU HMGA1 silencing restores normal stem cell characteristics in colon cancer stem cells by increasing p53 levels. Oncotarget (2014) 5:3234–45. 10.18632/oncotarget.1914 24833610 PMC4102806

[39] Ikushima H Todo T Ino Y Takahashi M Saito N Miyazawa K Glioma-initiating cells retain their tumorigenicity through integration of the Sox axis and Oct4 protein. J Biol Chem (2011) 286:41434–41. 10.1074/jbc.M111.300863 21987575 PMC3308855

[40] Favaro R Valotta M Ferri AL Latorre E Mariani J Giachino C Hippocampal development and neural stem cell maintenance require Sox2-dependent regulation of Shh. Nat Neurosci (2009) 12:1248–56. 10.1038/nn.2397 19734891

[41] Favaro R Appolloni I Pellegatta S Sanga AB Pagella P Gambini E Sox2 is required to maintain cancer stem cells in a mouse model of high-grade oligodendroglioma. Cancer Res (2014) 74:1833–44. 10.1158/0008-5472.CAN-13-1942 24599129

[42] Sunayama J Matsuda K Sato A Tachibana K Suzuki K Narita Y Crosstalk between the PI3K/mTOR and MEK/ERK pathways involved in the maintenance of self-renewal and tumorigenicity of glioblastoma stem-like cells. Stem Cells (2010) 28:1930–9. 10.1002/stem.521 20857497

[43] Emlet DR Gupta P Holgado-Madruga M Del Vecchio CA Mitra SS Han SY Targeting a glioblastoma cancer stem-cell population defined by EGF receptor variant III. Cancer Res (2014) 74:1238–49. 10.1158/0008-5472.CAN-13-1407 24366881 PMC5661963

[44] Yin J Park G Kim TH Hong JH Kim YJ Jin X Correction: pigment epithelium-derived factor (PEDF) expression induced by EGFRvIII promotes self-renewal and tumor progression of glioma stem cells. PLoS Biol (2016) 14:e1002367. 10.1371/journal.pbio.1002367 26751204 PMC4709175

[45] Baltus GA Kowalski MP Zhai H Tutter AV Quinn D Wall D Acetylation of sox2 induces its nuclear export in embryonic stem cells. Stem Cells (2009) 27:2175–84. 10.1002/stem.168 19591226

[46] Van Hoof D Muñoz J Braam SR Pinkse MW Linding R Heck AJ Phosphorylation dynamics during early differentiation of human embryonic stem cells. Cell Stem Cell (2009) 5:214–26. 10.1016/j.stem.2009.05.021 19664995

[47] Fang L Zhang L Wei W Jin X Wang P Tong Y A methylation-phosphorylation switch determines Sox2 stability and function in ESC maintenance or differentiation. Mol Cell (2014) 55:537–51. 10.1016/j.molcel.2014.06.018 25042802

[48] Fang X Yoon JG Li L Yu W Shao J Hua D The SOX2 response program in glioblastoma multiforme: an integrated ChIP-seq, expression microarray, and microRNA analysis. BMC Genomics (2011) 12:11. 10.1186/1471-2164-12-11 21211035 PMC3022822

[49] Berezovsky AD Poisson LM Cherba D Webb CP Transou AD Lemke NW Sox2 promotes malignancy in glioblastoma by regulating plasticity and astrocytic differentiation. Neoplasia (2014) 16:e119–25. 10.1016/j.neo.2014.03.006 24726753 PMC4094829

[50] Stupp R Mason WP van den Bent MJ Weller M Fisher B Taphoorn MJ Radiotherapy plus concomitant and adjuvant temozolomide for glioblastoma. N Engl J Med (2005) 352:987–96. 10.1056/NEJMoa043330 15758009

[51] Yang YP Chien Y Chiou GY Cherng JY Wang ML Lo WL Inhibition of cancer stem cell-like properties and reduced chemoradioresistance of glioblastoma using microRNA145 with cationic polyurethane-short branch PEI. Biomaterials (2012) 33:1462–76. 10.1016/j.biomaterials.2011.10.071 22098779

[52] Jeon HM Sohn YW Oh SY Kim SH Beck S Kim S ID4 imparts chemoresistance and cancer stemness to glioma cells by derepressing miR-9*-mediated suppression of SOX2. Cancer Res (2011) 71:3410–21. 10.1158/0008-5472.CAN-10-3340 21531766

[53] Arcella A Biagioni F Antonietta Oliva M Bucci D Frati A Esposito V Rapamycin inhibits the growth of glioblastoma. Brain Res (2013) 1495:37–51. 10.1016/j.brainres.2012.11.044 23261661

[54] Pachow D Wick W Gutmann DH Mawrin C. The mTOR signaling pathway as a treatment target for intracranial neoplasms. Neuro Oncol (2015) 17:189–99. 10.1093/neuonc/nou164 25165193 PMC4288522

[55] Soda Y Marumoto T Friedmann-Morvinski D Soda M Liu F Michiue H Transdifferentiation of glioblastoma cells into vascular endothelial cells. Proc Natl Acad Sci U S A (2011) 108:4274–80. 10.1073/pnas.1016030108 21262804 PMC3060261

[56] Scully S Francescone R Faibish M Bentley B Taylor SL Oh D Transdifferentiation of glioblastoma stem-like cells into mural cells drives vasculogenic mimicry in glioblastomas. J Neurosci (2012) 32:12950–60. 10.1523/JNEUROSCI.2017-12.2012 22973019 PMC3461581

[57] Brazel CY Limke TL Osborne JK Miura T Cai J Pevny L Sox2 expression defines a heterogeneous population of neurosphere-forming cells in the adult murine brain. Aging Cell (2005) 4:197–207. 10.1111/j.1474-9726.2005.00158.x 16026334

[58] Carrasco-Garcia E Arrizabalaga O Serrano M Lovell-Badge R Matheu A. Increased gene dosage of Ink4/Arf and p53 delays age-associated central nervous system functional decline. Aging Cell (2015) 14(4):710–4. 10.1111/acel.12343 25990896 PMC4531087

